# Shrinkage estimators of large covariance matrices with Toeplitz targets in array signal processing

**DOI:** 10.1038/s41598-022-21889-8

**Published:** 2022-11-08

**Authors:** Bin Zhang, Shoucheng Yuan

**Affiliations:** 1 College of Mathematics and Statistics, Guangxi Normal University, Guilin, 541004 Guangxi China; 2 College of Mathematics and Statistics, Pu’er University, Pu’er, 665000 Yunnan China

**Keywords:** Statistics, Electrical and electronic engineering

## Abstract

The problem of estimating a large covariance matrix arises in various statistical applications. This paper develops new covariance matrix estimators based on shrinkage regularization. Individually, we consider two kinds of Toeplitz-structured target matrices as the data come from the complex Gaussian distribution. We derive the optimal tuning parameter under the mean squared error criterion in closed form by discovering the mathematical properties of the two target matrices. We get some vital moment properties of the complex Wishart distribution, then simplify the optimal tuning parameter. By unbiasedly estimating the unknown scalar quantities involved in the optimal tuning parameter, we propose two shrinkage estimators available in the large-dimensional setting. For verifying the performance of the proposed covariance matrix estimators, we provide some numerical simulations and applications to array signal processing compared to some existing estimators.

## Introduction

The covariance matrix, which is a fundamental quantity for quantifying the variance and covariance among the variables, plays an essential role in both multivariate statistics and practical data processing^1, 2^. In general, people can hardly know the exact value of the covariance matrix. An overall experience is to find a well-conditioned covariance matrix estimator by the collected data^3–5^. In classic statistical theory with a low dimension and large sample size, the sample covariance matrix (SCM) is widely known as one standard estimator of the true covariance matrix. It enjoys lots of favorable statistical properties such as unbiasedness and consistency. Unfortunately, it suffers from either the small sample size or the large-dimension framework and needs to be improved^6, 7^.

During the last two decades, estimating the covariance matrix in large-dimension scenarios has attracted tremendous attention. Numerous regularization strategies have been designed to generate an high-performance covariance matrix estimator^8–13^. Among these, the linear shrinkage estimation is a widely-adopted regularization strategy to result in a well-conditioned covariance matrix estimator^14–17^. In detail, the shrinkage estimator consists of the SCM and a structured target matrix that represents prior information on the covariance structure. By carefully choosing the involved tuning parameter, also called the shrinkage intensity, the corresponding shrinkage estimator can significantly improve the SCM^18–20^. Although owning an analytical expression under the mean squared error (MSE) criterion, the optimal shrinkage estimator is unavailable in the practical data processing. The reason is the loss function in the MSE criterion evaluates the mean Frobenius distance between the true covariance matrix and the shrinkage estimator, resulting in the optimal shrinkage intensity containing some unknown scalar quantities concerning the true covariance matrix and the expectation operator. A direct approach to turning the optimal shrinkage estimator into an available one is the plug-in strategy, which replaces the unknown scalar quantities with their estimates^21–23^. It is not hard to understand that good estimates of the unknown scalar quantities will benefit both the corresponding available shrinkage intensity and the forthcoming available covariance matrix estimator^24, 25^.

A large proportion of shrinkage estimators is developed for dealing with large-dimensional data from the real number field. In^14^, the optimal shrinkage intensity is obtained for the spherical target. The concerned unknown scalar quantities are consistently estimated with small biases under the distribution-free setting. In^22, 24^, the unknown scalar quantities are respectively unbiasedly estimated under Gaussian and non-Gaussian distributions to develop shrinkage estimators with better performance. Furthermore, for the diagonal target and the tapered SCM target, the unknown scalar quantities in the corresponding optimal shrinkage intensities are respectively unbiasedly estimated to obtain high-performance covariance matrix estimators. What is worth mentioning is that the unknown scalar quantities corresponding to different target matrices are different. Moreover, estimating the unknown scalar quantities usually becomes difficult for the complicated target matrix. In^26^, for two kinds of Toeplitz-structured targets, the unknown scalar quantities are respectively unbiasedly estimated under Gaussian and non-Gaussian distributions. Furthermore, the shrinkage estimation is investigated under elliptically contoured distribution^27–29^.

In signal processing, the data usually comes from the complex number field. The covariance matrix is conjugate symmetric. The primary difference from the real number field is that the SCM with a multiplier follows the complex Wishart distribution. In^30^, the unknown scalar quantities are consistently estimated for one of the two Toeplitz-structured targets under the complex Gaussian distribution. However, the estimates of the involved are biased, leading a poor performance in a small sample size setting. In^31^, a non-parametric shrinkage estimator is developed via a proxy MSE under the complex Gaussian distribution. The advantage of this method is applying to any target matrix. However, the non-parametric shrinkage estimator is not optimal in the sense of MSE. In^32^, the optimal shrinkage estimator for the spherical target is established.

This paper investigates the shrinkage estimators or the two kinds of Toeplitz-structured targets under the complex Gaussian distribution. The main contributions are summarized as four-fold.

1. The optimal shrinkage intensities for the two kinds of Toeplitz-structured targets are obtained in the closed form under the MSE criterion.
2. We provide a useful moment property of the complex Wishart distribution for unbiasedly estimating the unknown scalar quantities involved in the optimal shrinkage intensities.
3. All the involved unknown scalar quantities are unbiasedly estimated under the complex Gaussian distribution. Then the corresponding available shrinkage estimators are immediately analytically expressed.
4. We provide some numerical simulations and applications to array signal processing to reveal the advantages of proposed covariance matrix estimators.

The remainder of this paper is as follows: In “Formulation”, we retrospect the shrinkage estimation of the covariance matrix as a quadratic optimization problem. “The optimal shrinkage intensity for Toeplitz-structured targets” provides the optimal shrinkage intensities and corresponding linear shrinkage estimators for the two kinds of Toeplitz-structured targets under complex Gaussian distribution. “Available shrinkage estimator for Toeplitz-structured targets” unbiasedly estimates the unknown scalar quantities involved in the optimal shrinkage estimators and proposes newly available shrinkage estimators. “Methods” provides some numerical simulations and two practical applications for exhibiting the advantages of proposed covariance matrix estimators. Section “Conclusion” concludes.

### Notations

The notation $\mathbb {R}$ represents the set of all real numbers. The notation $\mathbb {C}^m$ is the set of all *m*-dimensional complex column vectors, and $\mathbb {H}^n$ is the set of all $n \times n$ Hermitian matrices. The symbol $\mathbb {E}$ denotes the mathematical expectation. The bold symbol $\mathbf {0}$ and $\mathbf {1}$ respectively denote the column vectors having all entries 0 and 1 with an appropriate dimension. The symbol $\mathbf {I}_{n}$ denotes the $n \times n$ identity matrix. For a matrix, $\mathbf {A}$, $\mathbf {A}^{H}$, and $\Vert \mathbf {A}\Vert$ respectively denote its conjugate transpose and Frobenius matrix norm. For a squared matrix $\mathbf {A}$, $\mathbf {A}^{-1}$ and $\text {tr}(\mathbf {A})$ respectively denote its inverse and trace. For two real numbers *a* and *b*, $a \wedge b$ and $a \vee b$ respectively mean the maximum and minimum of *a* and *b*. For two positive integers *i*, *j*, $\delta _{ij}$ denotes the indicator function being 1 as $i=j$ or 0 otherwise.

## Formulation

Let $\mathbf {x}_1, \mathbf {x}_2, \dots , \mathbf {x}_n \in \mathbb {C}^p$ be an independent and identically distributed (i.i.d.) data drawn from the complex Gaussian distribution $\mathcal{CN}\mathcal{}(\varvec{\mathbf {0}}, \mathbf {\Sigma })$ with mean $\mathbf {0}$ and unknown covariance matrix $\mathbf {\Sigma }$. The task is estimating $\mathbf {\Sigma }$ by the available data $\mathbf {x}_i$. As is known to all, the SCM $\mathbf {S}= \frac{1}{n} \sum _{i=1}^{n} \mathbf {x}_{i} \mathbf {x}_{i}^{H}$ is one of standard covariance matrix estimators in the large sample scenario. However, it suffers from the curse of dimensionality in modern statistics. Furthermore, linear shrinkage regularization is a practical approach to obtaining a covariance matrix estimator with lower variance. Given a target matrix $\mathbf {T}\in \mathbb {H}^{p}$ represents an aspect prior information of covariance structure, the corresponding linear shrinkage estimator is expressed as a weighted combination between the SCM and the target, then we have

1 $$\begin{aligned} {\hat{\mathbf {\Sigma }}} = (1 - w) \mathbf {S}+ w \mathbf {T}\in \mathbb {H}^{p}, \end{aligned}$$

where $w \in \mathbb {R}$, the so-called shrinkage intensity, is an undetermined tuning parameter. For tuning the optimal parameter, we employ the MSE loss function

2 $$\begin{aligned} \mathcal {M}_{\mathbf {T}} (w) = \mathbb {E}[\Vert {\hat{\mathbf {\Sigma }}} - \mathbf {\Sigma }\Vert ^2] = \mathbb {E}[\Vert (1 - w) \mathbf {S}+ w \mathbf {T}- \mathbf {\Sigma }\Vert ^2]. \end{aligned}$$

Through a simple transformation, we have

3 $$\begin{aligned} \mathcal {M}_{\mathbf {T}} (w) = \mathbb {E}[\text {tr}(\mathbf {T}- \mathbf {S})^{2}] w^{2} - 2 \mathbb {E}[\text {tr}(\mathbf {T}- \mathbf {S})(\mathbf {\Sigma }- \mathbf {S})] w + c, \end{aligned}$$

where $c=\mathbb {E}[\text {tr}(\mathbf {S}- \mathbf {\Sigma })^{2}]$ is a constant. Hence, we can obtain the optimal shrinkage intensity in (3) by solving the following convex quadratic problem:

4 $$\begin{aligned} \begin{aligned} \min&\quad \mathbb {E}[\text {tr}(\mathbf {T}- \mathbf {S})^{2}] w^{2} - 2 \mathbb {E}[\text {tr}(\mathbf {T}- \mathbf {S})(\mathbf {\Sigma }- \mathbf {S})] w + c \\ \mathrm {s.t.}&\quad 0 \le w \le 1. \end{aligned} \end{aligned}$$

Then, the optimal shrinkage intensity for an arbitrary target matrix $\mathbf {T}$ is

5 $$\begin{aligned} w^{*} = 0 \wedge \frac{\mathbb {E}[\text {tr}(\mathbf {T}- \mathbf {S})(\mathbf {\Sigma }- \mathbf {S})]}{\mathbb {E}[\text {tr}(\mathbf {T}- \mathbf {S})^{2}]} \vee 1. \end{aligned}$$

## The optimal shrinkage intensity for Toeplitz-structured targets

Let $\mathbf {L}_p$ be the $p \times p$ matrix where the elements below the main diagonal equal 1 and 0 otherwise. For $q = -(p - 1), \dots , -1, 1, \dots , (p - 1)$, let $\mathbf {J}_q$ be the $p \times p$ matrix where the elements of *q*-th diagonal above (for $q > 0$) or below (for $q < 0$) main diagonal are equal to 1 and 0 otherwise. Then, the two types of Toeplitz-structured targets can be expressed as

6 $$\begin{aligned} \mathbf {T}_{1}&= \frac{\text {tr}(\mathbf {S})}{p} \mathbf {I}_p + \frac{2 \text {tr}(\mathbf {S}\mathbf {L}_p)}{ p (p - 1)} \mathbf {L}_p^{H} + \frac{2 \text {tr}(\mathbf {S}\mathbf {L}_p^{H})}{p (p - 1)} \mathbf {L}_p, \end{aligned}$$

7 $$\begin{aligned} \mathbf {T}_{2}&= \sum ^{p - 1}_{q = -(p - 1)} \frac{\text {tr}(\mathbf {S}\mathbf {J}_q)}{p - |q|} \mathbf {J}_q^H. \end{aligned}$$

Both $\mathbf {T}_{1}$ and $\mathbf {T}_{2}$ are Hermitian and Toeplitz-structured matrices. Furthermore, they have the following property.

### Proposition 1

For $i = 1, 2$, the following equality about the target $\mathbf {T}_{i}$ and the SCM holds:

8 $$\begin{aligned} \text {tr}(\mathbf {T}_{i} \mathbf {S}) = \text {tr}(\mathbf {T}_{i}^{2}). \end{aligned}$$

### Proof

When the target matrix is $\mathbf {T}_{1}$, we have

9 $$\begin{aligned} \text {tr}(\mathbf {T}_{1} \mathbf {S})&= \text {tr}\left( \frac{\text {tr}(\mathbf {S})}{p} \mathbf {S}+ \frac{2 \text {tr}(\mathbf {S}\mathbf {L}_{p})}{p(p-1)} \mathbf {L}_{p}^{H}\mathbf {S}+ \frac{2 \text {tr}(\mathbf {S}\mathbf {L}_{p}^{H})}{p(p-1)} \mathbf {L}_{p}\mathbf {S}\right) \nonumber \\&= \frac{1}{p} \text {tr}^{2}(\mathbf {S}) + \frac{2}{p(p-1)} \text {tr}(\mathbf {S}\mathbf {L}_{p}) \text {tr}(\mathbf {L}_{p}^{H} \mathbf {S}) + \frac{2}{p(p-1)} \text {tr}(\mathbf {S}\mathbf {L}_{p}^{H}) \text {tr}(\mathbf {L}_{p} \mathbf {S}) \nonumber \\&= \frac{1}{p} \text {tr}^{2}(\mathbf {S}) + \frac{4}{p(p-1)} \text {tr}(\mathbf {S}\mathbf {L}_{p}) \text {tr}(\mathbf {S}\mathbf {L}_{p}^{H}), \end{aligned}$$

and

10 $$\begin{aligned} \text {tr}(\mathbf {T}_{1}^{2})&= \text {tr}\left( \frac{\text {tr}(\mathbf {S})}{p} \mathbf {I}_{p} + \frac{2 \text {tr}(\mathbf {S}\mathbf {L}_{p})}{p(p-1)} \mathbf {L}_{p}^{H} + \frac{2 \text {tr}(\mathbf {S}\mathbf {L}_{p}^{H})}{p(p-1)} \mathbf {L}_{p} \right) ^{2} \nonumber \\&= \frac{1}{p} \text {tr}^{2}(\mathbf {S}) + \frac{4}{p^{2}(p-1)^{2}} \text {tr}^{2}(\mathbf {S}\mathbf {L}_{p}) \text {tr}(\mathbf {L}_{p}^{H} \mathbf {L}_{p}^{H}) + \frac{4}{p^{2}(p-1)^{2}} \text {tr}^{2}(\mathbf {S}\mathbf {L}_{p}^{H}) \text {tr}(\mathbf {L}_{p} \mathbf {L}_{p}) \nonumber \\&\quad + \frac{4}{p^{2}(p-1)} \text {tr}(\mathbf {S}) \text {tr}(\mathbf {S}\mathbf {L}_{p}) \text {tr}(\mathbf {L}_{p}^{H}) + \frac{4}{p^{2}(p-1)} \text {tr}(\mathbf {S}) \text {tr}(\mathbf {S}\mathbf {L}_{p}^{H}) \text {tr}(\mathbf {L}_{p}) \nonumber \\&\quad + \frac{8}{p^{2}(p-1)^{2}} \text {tr}(\mathbf {S}\mathbf {L}_{p}) \text {tr}(\mathbf {S}\mathbf {L}_{p}^{H}) \text {tr}(\mathbf {L}_{p}^{H} \mathbf {L}_{p}). \end{aligned}$$

Moreover, for the matrix $\mathbf {L}_{p}$, we have $\text {tr}(\mathbf {L}_{p}) = 0$, $\text {tr}(\mathbf {L}_{p}^{H}) = 0$, $\text {tr}(\mathbf {L}_{p} \mathbf {L}_{p}) = 0$, $\text {tr}(\mathbf {L}_{p}^{H} \mathbf {L}_{p}^{H}) = 0$ and $\text {tr}(\mathbf {L}_{p}^{H} \mathbf {L}_{p}) = \frac{p(p-1)}{2}$. By substituting these equalities about $\mathbf {L}_{p}$ into (9) and (10), we can obtain

11 $$\begin{aligned} \text {tr}(\mathbf {T}_{1}^{2}) = \frac{1}{p} \text {tr}^{2}(\mathbf {S}) + \frac{4}{p(p-1)} \text {tr}(\mathbf {S}\mathbf {L}_{p}) \text {tr}(\mathbf {S}\mathbf {L}_{p}^{H}) = \text {tr}(\mathbf {T}_{1} \mathbf {S}). \end{aligned}$$

For the target matrix $\mathbf {T}_{2}$, we have

12 $$\begin{aligned} \text {tr}(\mathbf {T}_{2}^{2}) = \text {tr}(\mathbf {T}_{2} \mathbf {S}) = \sum _{q=-(p-1)}^{p-1} \frac{1}{p-|q|} \text {tr}(\mathbf {S}\mathbf {J}_{q}) \text {tr}(\mathbf {S}\mathbf {J}_{q}^{H}). \end{aligned}$$

Therefore, the equality (8) holds for $\mathbf {T}_{1}$ and $\mathbf {T}_{2}$.

### Remark 1

It is not hard to verify that the relationship given by (8) also holds for the spherical target $\mathbf {T}_{3} = \frac{\text {tr}(\mathbf {S})}{p} \mathbf {I}_{p}$ and the diagonal target $\mathbf {T}_{4} = \text {diag}(s_{11}, \dots , s_{pp})$, which are two popular target matrices in linear shrinkage estimation.

By Proposition 1, when the target is $\mathbf {T}= \mathbf {T}_{1}$ or $\mathbf {T}= \mathbf {T}_{2}$, the optimal shrinkage intensity in (5) turns to be

13 $$\begin{aligned} w^{*} = 0 \wedge \left( 1 - \frac{\text {tr}(\mathbf {\Sigma }^{2}) - \mathbb {E}[\text {tr}(\mathbf {T}\mathbf {\Sigma })]}{\mathbb {E}[\text {tr}(\mathbf {S}^{2})] - \mathbb {E}[\text {tr}(\mathbf {T}^{2})]} \right) \vee 1. \end{aligned}$$

Furthermore, under complex Gaussian distribution, we have the following results.

### Theorem 2

Assume $\mathbf {x}_1, \mathbf {x}_2, \dots , \mathbf {x}_n \in \mathbb {C}^p$ is an i.i.d. sample from $\mathcal{CN}\mathcal{}(\varvec{\mathbf {0}}, \mathbf {\Sigma })$, $\mathbf {A}$ is an arbitrary nonrandom $p \times p$ matrix, $\mathbf {S}$ is the sample covariance matrix, the following relationships hold:

14 $$\begin{aligned} \mathbb {E}[\text {tr}(\mathbf {S}\mathbf {A}\mathbf {S}\mathbf {A}^{H})]&= \text {tr}(\mathbf {\Sigma }\mathbf {A}\mathbf {\Sigma }\mathbf {A}^{H}) + \frac{1}{n} \text {tr}(\mathbf {\Sigma }\mathbf {A}) \text {tr}(\mathbf {\Sigma }\mathbf {A}^{H}), \end{aligned}$$

15 $$\begin{aligned} \mathbb {E}[\text {tr}(\mathbf {S}\mathbf {A}) \text {tr}(\mathbf {S}\mathbf {A}^{H})]&= \frac{1}{n} \text {tr}(\mathbf {\Sigma }\mathbf {A}\mathbf {\Sigma }\mathbf {A}^{H}) + \text {tr}(\mathbf {\Sigma }\mathbf {A}) \text {tr}(\mathbf {\Sigma }\mathbf {A}^{H}). \end{aligned}$$

### Proof

Because the i.i.d. sample $\mathbf {x}_1, \mathbf {x}_2, \dots , \mathbf {x}_n \in \mathbb {C}^p$ comes from the complex Gaussian distribution $\mathcal{CN}\mathcal{}(\mathbf {0}, \mathbf {\Sigma })$, then $n \mathbf {S}$ follows the complex Wishart distribution $\mathcal{CW}\mathcal{}(\mathbf {\Sigma }, n)$, where *n* is the degree of freedom. For an arbitrary nonrandom $p \times p$ matrix $\mathbf {A}$, we have

16 $$\begin{aligned} \mathbb {E}[\mathbf {S}\mathbf {A}\mathbf {S}\mathbf {A}^{H}] = \mathbf {\Sigma }\mathbf {A}\mathbf {\Sigma }\mathbf {A}^{H} + \frac{1}{n} \text {tr}(\mathbf {\Sigma }\mathbf {A}) \mathbf {\Sigma }\mathbf {A}^{H}. \end{aligned}$$

Then, we can obtain

17 $$\begin{aligned} \mathbb {E}[\text {tr}(\mathbf {S}\mathbf {A}\mathbf {S}\mathbf {A}^{H})] = \text {tr}(\mathbf {\Sigma }\mathbf {A}\mathbf {\Sigma }\mathbf {A}^{H}) + \frac{1}{n} \text {tr}(\mathbf {\Sigma }\mathbf {A}) \text {tr}(\mathbf {\Sigma }\mathbf {A}^{H}). \end{aligned}$$

Next, we compute $\mathbb {E}[\text {tr}(\mathbf {S}\mathbf {A}) \text {tr}(\mathbf {S}\mathbf {A}^{H})]$ under complex Gaussian distribution. Denote $\mathbf {A}= (a_{ij})_{p\times p}$, then we have $\mathbf {A}^{H} = (a_{ji}^{\dagger })_{p\times p}$. For a random matrix $\mathbf {W}= (w_{ij})_{p\times p}$, we have

18 $$\begin{aligned} \text {tr}(\mathbf {W}\mathbf {A}) = \sum _{i=1}^{p} \sum _{j=1}^{p} w_{ij} a_{ji}, \quad \text {tr}(\mathbf {W}\mathbf {A}^{H}) = \sum _{i=1}^{p} \sum _{j=1}^{p} w_{ij} a_{ij}^{\dagger }. \end{aligned}$$

Therefore, we have

19 $$\begin{aligned} \text {tr}(\mathbf {W}\mathbf {A}) \text {tr}(\mathbf {W}\mathbf {A}^{H}) = \sum _{i=1}^{p} \sum _{j=1}^{p} \sum _{k=1}^{p} \sum _{l=1}^{p} a_{ji} a_{kl}^{\dagger } w_{ij} w_{kl}. \end{aligned}$$

When $\mathbf {W}$ follows the complex Wishart distribution $\mathcal{CW}\mathcal{}(\mathbf {I}_{p}, n)$, we have

20 $$\begin{aligned} \mathbb {E}[\text {tr}(\mathbf {W}\mathbf {A}) \text {tr}(\mathbf {W}\mathbf {A}^{H})]&= \sum _{i=1}^{p} \sum _{j=1}^{p} \sum _{k=1}^{p} \sum _{l=1}^{p} a_{ji} a_{kl}^{\dagger } \mathbb {E}[w_{ij} w_{kl}] \nonumber \\&= \sum _{i=1}^{p} \sum _{j=1}^{p} \sum _{k=1}^{p} \sum _{l=1}^{p} a_{ji} a_{kl}^{\dagger } (b_{1} \delta _{ij} \delta _{kl} + b_{2} \delta _{il} \delta _{jk}) \nonumber \\&= \sum _{i=1}^{p} \sum _{k=1}^{p} b_{1} a_{ii} a_{kk}^{\dagger } + \sum _{i=1}^{p} \sum _{k=1}^{p} b_{2} a_{ji} a_{ji}^{\dagger } \nonumber \\&= b_{1} \text {tr}(\mathbf {A}) \text {tr}(\mathbf {A}^{H}) + b_{2} \text {tr}(\mathbf {A}\mathbf {A}^{H}), \end{aligned}$$

where $\delta _{ij}$ is the indicator function which equals 1 when $i=j$ or 0 otherwise, $b_{1}$ and $b_{2}$ are two undetermined constants. Furthermore, when $\mathbf {W}$ follows the complex Wishart distribution $\mathcal{CW}\mathcal{}(\mathbf {\Sigma }, n)$, we have $\mathbf {G}^{-1} \mathbf {W}\mathbf {G}^{-H} \sim \mathcal{CW}\mathcal{}(\mathbf {I}_{p}, n)$ by denoting $\mathbf {\Sigma }= \mathbf {G}\mathbf {G}^{H}$. Hence, for an arbitrary nonrandom $p \times p$ matrix $\mathbf {A}$, we can obtain

21 $$\begin{aligned}&\mathbb {E}[\text {tr}(\mathbf {G}^{-1} \mathbf {W}\mathbf {G}^{-H} \mathbf {B}) \text {tr}(\mathbf {G}^{-1} \mathbf {W}\mathbf {G}^{-H} \mathbf {B}^{H})] \nonumber \\&= \mathbb {E}[\text {tr}(\mathbf {W}\mathbf {G}^{-H} \mathbf {B}\mathbf {G}^{-1} ) \text {tr}(\mathbf {W}\mathbf {G}^{-H} \mathbf {B}^{H} \mathbf {G}^{-1} )] \nonumber \\&= b_{1} \text {tr}(\mathbf {B}) \text {tr}(\mathbf {B}^{H}) + b_{2} \text {tr}(\mathbf {B}\mathbf {B}^{H}). \end{aligned}$$

Denote $\mathbf {A}= \mathbf {G}^{-H} \mathbf {B}\mathbf {G}^{-1}$, then $\mathbf {B}= \mathbf {G}^{H} \mathbf {A}\mathbf {G}$. Therefore, we have

22 $$\begin{aligned} \mathbb {E}[\text {tr}(\mathbf {W}\mathbf {A}) \text {tr}(\mathbf {W}\mathbf {A}^{H})]&= b_{1} \text {tr}(\mathbf {G}^{H} \mathbf {A}\mathbf {G}) \text {tr}(\mathbf {G}^{H} \mathbf {A}^{H} \mathbf {G}) + b_{2} \text {tr}(\mathbf {G}^{H} \mathbf {A}\mathbf {G}\mathbf {G}^{H} \mathbf {A}^{H} \mathbf {G}) \nonumber \\&= b_{1} \text {tr}(\mathbf {G}\mathbf {G}^{H} \mathbf {A}) \text {tr}(\mathbf {G}\mathbf {G}^{H} \mathbf {A}^{H}) + b_{2} \text {tr}(\mathbf {G}\mathbf {G}^{H} \mathbf {A}\mathbf {G}\mathbf {G}^{H} \mathbf {A}^{H}) \nonumber \\&= b_{1} \text {tr}(\mathbf {\Sigma }\mathbf {A}) \text {tr}(\mathbf {\Sigma }\mathbf {A}^{H}) + b_{2} \text {tr}(\mathbf {\Sigma }\mathbf {A}\mathbf {\Sigma }\mathbf {A}^{H}). \end{aligned}$$

Next, we turn to determine the constants $b_{1}$ and $b_{2}$. Noticing that (22) holds for arbitrary $\mathbf {\Sigma }$ and $\mathbf {A}$, we can plug some specific matrices into (22) to obtain $b_{1}$ and $b_{2}$. Firstly, let $\mathbf {\Sigma }= \mathbf {I}_{p}$, $\mathbf {A}= (a_{i j})_{p \times p}$ with $a_{i j} = 1$ if $i = j = 1$ and $a_{i j} = 0$ if else, then we have

23 $$\begin{aligned} \mathbb {E}[w_{11}^{2}] = b_{1} + b_{2}. \end{aligned}$$

Secondly, let $\mathbf {\Sigma }= \mathbf {I}_{p}$ and $\mathbf {A}= \mathbf {I}_{p}$, we have

24 $$\begin{aligned} \sum _{i=1}^{p} \sum _{j=1}^{p} \mathbb {E}[w_{ii} w_{jj}] = b_{1} p^{2} + b_{2} p. \end{aligned}$$

For $i = 1, \dots , p$, $w_{ii}$ is the sum of squares of 2*n* i.i.d. Gaussian random variables with variance $\frac{1}{2}$. Hence, we can obtain that $2 w_{ii} \sim \chi ^{2} (2n)$. By the moment property of $\chi ^{2}$ distribution, we have $\mathbb {E}[w_{ii}] = n$, $\mathbb {E}[w_{ii}^{2}] = n(n+1)$. Therefore, we have

25 $$\begin{aligned} \sum _{i=1}^{p} \sum _{j=1}^{p}\mathbb {E}[w_{ii} w_{jj}]&= \sum _{i=1}^{p} \mathbb {E}[w_{ii}^{2}] + \sum _{i \ne j} \mathbb {E}[w_{ii} w_{jj}] \nonumber \\&= \sum _{i=1}^{p} \mathbb {E}[w_{ii}^{2}] + \sum _{i \ne j} \mathbb {E}[w_{ii}] \mathbb {E}[w_{jj}] \nonumber \\&= n(n+1)p + p(p-1)n^{2}. \end{aligned}$$

Then, the equalities (23) and (24) becomes

26 $$\begin{aligned} b_{1} + b_{2}&= n(n+1), \end{aligned}$$

27 $$\begin{aligned} b_{1} p^{2} + b_{2} p&= n(n+1)p + p(p-1)n^{2}. \end{aligned}$$

By solving the above system of equations, we can easily obtain $b_{1} = n^{2}$, $b_{2} = n$. Therefore, we have

28 $$\begin{aligned} \mathbb {E}[\text {tr}(\mathbf {W}\mathbf {A}) \text {tr}(\mathbf {W}\mathbf {A}^{H})] = n^{2} \text {tr}(\mathbf {\Sigma }\mathbf {A}) \text {tr}(\mathbf {\Sigma }\mathbf {A}^{H}) + n \text {tr}(\mathbf {\Sigma }\mathbf {A}\mathbf {\Sigma }\mathbf {A}^{H}). \end{aligned}$$

Due to $n \mathbf {S}\sim \mathcal{CW}\mathcal{}(\mathbf {\Sigma }, n)$, we have

29 $$\begin{aligned} \mathbb {E}[\text {tr}(n \mathbf {S}\mathbf {A}) \text {tr}(n \mathbf {S}\mathbf {A}^{H})] = n^{2} \text {tr}(\mathbf {\Sigma }\mathbf {A}) \text {tr}(\mathbf {\Sigma }\mathbf {A}^{H}) + n \text {tr}(\mathbf {\Sigma }\mathbf {A}\mathbf {\Sigma }\mathbf {A}^{H}). \end{aligned}$$

By dividing $n^{2}$, we have

30 $$\begin{aligned} \mathbb {E}[\text {tr}(\mathbf {S}\mathbf {A}) \text {tr}(\mathbf {S}\mathbf {A}^{H})] = \frac{1}{n} \text {tr}(\mathbf {\Sigma }\mathbf {A}\mathbf {\Sigma }\mathbf {A}^{H}) + \text {tr}(\mathbf {\Sigma }\mathbf {A}) \text {tr}(\mathbf {\Sigma }\mathbf {A}^{H}). \end{aligned}$$

It is worth noticing that Theorem 2 holds for arbitrary nonrandom complex matrix $\mathbf {A}$. As $\mathbf {A}= \mathbf {I}_{p}$, we can obtain

31 $$\begin{aligned} \mathbb {E}[\text {tr}(\mathbf {S}^{2})]&= \text {tr}(\mathbf {\Sigma }^{2}) + \frac{1}{n} \text {tr}^{2}(\mathbf {\Sigma }), \end{aligned}$$

32 $$\begin{aligned} \mathbb {E}[\text {tr}^{2} (\mathbf {S})]&= \frac{1}{n} \text {tr}(\mathbf {\Sigma }^{2}) + \text {tr}^{2}(\mathbf {\Sigma }). \end{aligned}$$

Therefore, the expressions of $\mathbb {E}[\text {tr}(\mathbf {S}^{2})]$ under Gaussian distribution and complex Gaussian distribution are different from each other. The same to $\mathbb {E}[\text {tr}^{2} (\mathbf {S})]$. As $\mathbf {A}= \mathbf {L}_{p}$, we can obtain

33 $$\begin{aligned} \mathbb {E}[\text {tr}(\mathbf {S}\mathbf {L}_{p} \mathbf {S}\mathbf {L}_{p}^{H})]&= \text {tr}(\mathbf {\Sigma }\mathbf {L}_{p} \mathbf {\Sigma }\mathbf {L}_{p}^{H}) + \frac{1}{n} \text {tr}(\mathbf {\Sigma }\mathbf {L}_{p}) \text {tr}(\mathbf {\Sigma }\mathbf {L}_{p}^{H}), \end{aligned}$$

34 $$\begin{aligned} \mathbb {E}[\text {tr}(\mathbf {S}\mathbf {L}_{p}) \text {tr}(\mathbf {S}\mathbf {L}_{p}^{H})]&= \frac{1}{n} \text {tr}(\mathbf {\Sigma }\mathbf {L}_{p} \mathbf {\Sigma }\mathbf {L}_{p}^{H}) + \text {tr}(\mathbf {\Sigma }\mathbf {L}_{p}) \text {tr}(\mathbf {\Sigma }\mathbf {L}_{p}^{H}). \end{aligned}$$

Moreover, for $q = -(p-1), \dots , p-1$ and $\mathbf {A}= \mathbf {J}_{q}$, we can obtain

35 $$\begin{aligned} \mathbb {E}[\text {tr}(\mathbf {S}\mathbf {J}_{q} \mathbf {S}\mathbf {J}_{q}^{H})]&= \text {tr}(\mathbf {\Sigma }\mathbf {J}_{q} \mathbf {\Sigma }\mathbf {J}_{q}^{H}) + \frac{1}{n} \text {tr}(\mathbf {\Sigma }\mathbf {J}_{q}) \text {tr}(\mathbf {\Sigma }\mathbf {J}_{q}^{H}), \end{aligned}$$

36 $$\begin{aligned} \mathbb {E}[\text {tr}(\mathbf {S}\mathbf {J}_{q}) \text {tr}(\mathbf {S}\mathbf {J}_{q}^{H})]&= \frac{1}{n} \text {tr}(\mathbf {\Sigma }\mathbf {J}_{q} \mathbf {\Sigma }\mathbf {J}_{q}^{H}) + \text {tr}(\mathbf {\Sigma }\mathbf {J}_{q}) \text {tr}(\mathbf {\Sigma }\mathbf {J}_{q}^{H}). \end{aligned}$$

### Theorem 3

Assume $\mathbf {x}_1, \mathbf {x}_2, \dots , \mathbf {x}_n \in \mathbb {C}^p$ is an i.i.d. sample from $\mathcal{CN}\mathcal{}(\varvec{\mathbf {0}}, \mathbf {\Sigma })$, the optimal shrinkage intensity for $\mathbf {T}_{1}$ is

37 $$\begin{aligned} w_{\mathbf {T}_{1}}^{*} = 0 \wedge \frac{\text {tr}^{2}(\mathbf {\Sigma }) - \frac{1}{p}\text {tr}(\mathbf {\Sigma }^{2}) - \frac{4}{p(p-1)} \text {tr}(\mathbf {\Sigma }\mathbf {L}_{p} \mathbf {\Sigma }\mathbf {L}_{p}^{H})}{\text {tr}^{2}(\mathbf {\Sigma }) - \frac{1}{p}\text {tr}(\mathbf {\Sigma }^{2}) - \frac{4}{p(p-1)} \text {tr}(\mathbf {\Sigma }\mathbf {L}_{p} \mathbf {\Sigma }\mathbf {L}_{p}^{H}) + d_{\mathbf {T}_{1}}} \vee 1, \end{aligned}$$

where

38 $$\begin{aligned} d_{\mathbf {T}_{1}} = n \text {tr}(\mathbf {\Sigma }^{2}) - \frac{n}{p} \text {tr}^{2} (\mathbf {\Sigma }) - \frac{4n}{p(p-1)} \text {tr}(\mathbf {\Sigma }\mathbf {L}_{p})\text {tr}(\mathbf {\Sigma }\mathbf {L}_{p}^{H}). \end{aligned}$$

The optimal shrinkage intensity for $\mathbf {T}_{2}$ is

39 $$\begin{aligned} w_{\mathbf {T}_{2}}^{*} = 0 \wedge \frac{\text {tr}^{2}(\mathbf {\Sigma }) - \sum _{q=-(p-1)}^{p-1} \frac{1}{p-|q|} \text {tr}(\mathbf {\Sigma }\mathbf {J}_{q} \mathbf {\Sigma }\mathbf {J}_{q}^{H})}{\text {tr}^{2}(\mathbf {\Sigma }) - \sum _{q=-(p-1)}^{p-1} \frac{1}{p-|q|} \text {tr}(\mathbf {\Sigma }\mathbf {J}_{q} \mathbf {\Sigma }\mathbf {J}_{q}^{H}) + d_{\mathbf {T}_{2}}} \vee 1, \end{aligned}$$

where

40 $$\begin{aligned} d_{\mathbf {T}_{2}} = n \text {tr}(\mathbf {\Sigma }^{2}) - \sum _{q=-(p-1)}^{p-1} \frac{n}{p-|q|} \text {tr}(\mathbf {\Sigma }\mathbf {J}_{q}) \text {tr}(\mathbf {\Sigma }\mathbf {J}_{q}^{H}). \end{aligned}$$

### Proof

By the optimal shrinkage intensity given by (13), we need to compute $\mathbb {E}[\text {tr}(\mathbf {S}^{2})]$, $\mathbb {E}[\text {tr}(\mathbf {T}\mathbf {\Sigma })]$ and $\mathbb {E}[\text {tr}(\mathbf {T}^{2})]$ under complex Gaussian distribution. By Eq. (14), we have

41 $$\begin{aligned} \mathbb {E}[\text {tr}(\mathbf {S}^{2})] = \frac{1}{n} \text {tr}^{2}(\mathbf {\Sigma }) + \text {tr}(\mathbf {\Sigma }^{2}). \end{aligned}$$

Next, we just need to compute $\mathbb {E}[\text {tr}(\mathbf {T}\mathbf {\Sigma })]$ and $\mathbb {E}[\text {tr}(\mathbf {T}^{2})]$. When the target is $\mathbf {T}_{1}$, we have

42 $$\begin{aligned} \mathbb {E}[\text {tr}(\mathbf {T}\mathbf {\Sigma })]&= \mathbb {E}\left[ \text {tr}\left( \frac{\text {tr}(\mathbf {S})}{p} \mathbf {\Sigma }+ \frac{2 \text {tr}(\mathbf {S}\mathbf {L}_{p})}{p(p-1)} \mathbf {L}_{p}^{H} \mathbf {\Sigma }+ \frac{2 \text {tr}(\mathbf {S}\mathbf {L}_{p}^{H})}{p(p-1)} \mathbf {L}_{p} \mathbf {\Sigma }\right) \right] \nonumber \\&= \frac{1}{p} \text {tr}^{2}(\mathbf {\Sigma }) + \frac{2}{p(p-1)} \text {tr}(\mathbf {\Sigma }\mathbf {L}_{p}) \text {tr}(\mathbf {L}_{p}^{H} \mathbf {\Sigma }) + \frac{2}{p(p-1)} \text {tr}(\mathbf {\Sigma }\mathbf {L}_{p}^{H}) \text {tr}(\mathbf {L}_{p} \mathbf {\Sigma }) \nonumber \\&= \frac{1}{p} \text {tr}^{2}(\mathbf {\Sigma }) + \frac{4}{p(p-1)} \text {tr}(\mathbf {\Sigma }\mathbf {L}_{p}) \text {tr}(\mathbf {\Sigma }\mathbf {L}_{p}^{H}). \end{aligned}$$

By Eq. (11), we have

43 $$\begin{aligned} \mathbb {E}[\text {tr}(\mathbf {T}^{2})] = \mathbb {E}[\text {tr}(\mathbf {T}_{1} \mathbf {S})] = \frac{1}{p} \mathbb {E}[\text {tr}^{2}(\mathbf {S})] + \frac{4}{p(p-1)} \mathbb {E}[\text {tr}(\mathbf {S}\mathbf {L}_{p}) \text {tr}(\mathbf {S}\mathbf {L}_{p}^{H})]. \end{aligned}$$

By Eqs. (33) and (34), we have

44 $$\begin{aligned}&\mathbb {E}[\text {tr}(\mathbf {T}^{2})] \nonumber \\&= \frac{1}{p} \left( \frac{1}{n} \text {tr}(\mathbf {\Sigma }^{2}) + \text {tr}^{2}(\mathbf {\Sigma }) \right) + \frac{4}{p(p-1)} \left( \frac{1}{n} \text {tr}(\mathbf {\Sigma }\mathbf {L}_{p} \mathbf {\Sigma }\mathbf {L}_{p}^{H}) + \text {tr}(\mathbf {\Sigma }\mathbf {L}_{p}) \text {tr}(\mathbf {\Sigma }\mathbf {L}_{p}^{H}) \right) \nonumber \\&= \frac{1}{np} \text {tr}(\mathbf {\Sigma }^{2}) + \frac{1}{p} \text {tr}^{2}(\mathbf {\Sigma }) + \frac{4}{np(p-1)} \text {tr}(\mathbf {\Sigma }\mathbf {L}_{p} \mathbf {\Sigma }\mathbf {L}_{p}^{H}) + \frac{4}{p(p-1)} \text {tr}(\mathbf {\Sigma }\mathbf {L}_{p}) \text {tr}(\mathbf {\Sigma }\mathbf {L}_{p}^{H}). \end{aligned}$$

By substituting (41), (42) and (44) into (13), the optimal shrinkage intensity becomes

45 $$\begin{aligned} w_{\mathbf {T}_{1}}^{*} = 0 \wedge \frac{\frac{1}{n} \text {tr}^{2}(\mathbf {\Sigma }) - \frac{1}{np}\text {tr}(\mathbf {\Sigma }^{2}) - \frac{4}{np(p-1)} \text {tr}(\mathbf {\Sigma }\mathbf {L}_{p} \mathbf {\Sigma }\mathbf {L}_{p}^{H})}{\frac{1}{n} \text {tr}^{2}(\mathbf {\Sigma }) - \frac{1}{np}\text {tr}(\mathbf {\Sigma }^{2}) - \frac{4}{np(p-1)} \text {tr}(\mathbf {\Sigma }\mathbf {L}_{p} \mathbf {\Sigma }\mathbf {L}_{p}^{H}) + d_{\mathbf {T}_{1}}} \vee 1, \end{aligned}$$

where $$d_{\mathbf {T}_{1}} = \text {tr}(\mathbf {\Sigma }^{2}) - \frac{1}{p} \text {tr}^{2} (\mathbf {\Sigma }) - \frac{4}{p(p-1)} \text {tr}(\mathbf {\Sigma }\mathbf {L}_{p})\text {tr}(\mathbf {\Sigma }\mathbf {L}_{p}^{H})$$.

In the same manner, when the target is $\mathbf {T}_{2}$, we have

46 $$\begin{aligned} \mathbb {E}[\text {tr}(\mathbf {T}\mathbf {\Sigma })]&= \mathbb {E}\left[ \text {tr}\left( \sum _{q=-(p-1)}^{p-1} \frac{\text {tr}(\mathbf {S}\mathbf {J}_{q})}{p-|q|} \mathbf {J}_{q}^{H} \mathbf {\Sigma }\right) \right] \nonumber \\&= \sum _{q=-(p-1)}^{p-1} \frac{1}{p-|q|} \text {tr}(\mathbf {\Sigma }\mathbf {J}_{q}) \text {tr}(\mathbf {\Sigma }\mathbf {J}_{q}^{H}), \end{aligned}$$

and

47 $$\begin{aligned} \mathbb {E}[\text {tr}(\mathbf {T}\mathbf {S})] = \mathbb {E}[\text {tr}(\mathbf {T}^{2})] = \sum _{q=-(p-1)}^{p-1} \frac{1}{p-|q|} \mathbb {E}[\text {tr}(\mathbf {S}\mathbf {J}_{q}) \text {tr}(\mathbf {S}\mathbf {J}_{q}^{H})]. \end{aligned}$$

By Eq. (36), we can obtain

48 $$\begin{aligned} \mathbb {E}[\text {tr}(\mathbf {T}^{2})] = \sum _{q=-(p-1)}^{p-1} \frac{1}{p-|q|} \left( \frac{1}{n} \text {tr}(\mathbf {\Sigma }\mathbf {J}_{q} \mathbf {\Sigma }\mathbf {J}_{q}^{H}) + \text {tr}(\mathbf {\Sigma }\mathbf {J}_{q}) \text {tr}(\mathbf {\Sigma }\mathbf {J}_{q}^{H}) \right) . \end{aligned}$$

By substituting (41), (46) and (48) into (13), the optimal shrinkage intensity becomes

49 $$\begin{aligned} w_{\mathbf {T}_{2}}^{*} = 0 \wedge \frac{\frac{1}{n} \text {tr}^{2}(\mathbf {\Sigma }) - \frac{1}{n} \sum _{q=-(p-1)}^{p-1} \frac{1}{p-|q|} \text {tr}(\mathbf {\Sigma }\mathbf {J}_{q} \mathbf {\Sigma }\mathbf {J}_{q}^{H})}{\frac{1}{n} \text {tr}^{2}(\mathbf {\Sigma }) - \frac{1}{n} \sum _{q=-(p-1)}^{p-1} \frac{1}{p-|q|} \text {tr}(\mathbf {\Sigma }\mathbf {J}_{q} \mathbf {\Sigma }\mathbf {J}_{q}^{H}) + d_{\mathbf {T}_{2}}} \vee 1, \end{aligned}$$

where $$d_{\mathbf {T}_{2}} = \text {tr}(\mathbf {\Sigma }^{2}) - \sum _{q=-(p-1)}^{p-1} \frac{1}{p-|q|} \text {tr}(\mathbf {\Sigma }\mathbf {J}_{q}) \text {tr}(\mathbf {\Sigma }\mathbf {J}_{q}^{H})$$.

We remind that the optimal shrinkage intensities given by (37) and (39) involve the true covariance matrix and are unavailable in real applications. Even so, the theoretical optimal tuning parameters are still crucial in shrinkage estimation and can be employed to evaluate the available ones.

## Available shrinkage estimator for Toeplitz-structured targets

In this section, we estimate the unknown scalar quantities in (37) and (39), and obtain available shrinkage intensities by plug-in strategy.

### Corollary 4

Let $\mathbf {x}_1, \mathbf {x}_2, \dots , \mathbf {x}_n \in \mathbb {C}^p$ be an i.i.d. sample from $\mathcal{CN}\mathcal{}(\varvec{\mathbf {0}}, \mathbf {\Sigma })$. For an arbitrary nonrandom matrix $\mathbf {A}$, the unbiased estimates of $\text {tr}(\mathbf {\Sigma }\mathbf {A}\mathbf {\Sigma }\mathbf {A}^{H})$ and $\text {tr}(\mathbf {\Sigma }\mathbf {A}) \text {tr}(\mathbf {\Sigma }\mathbf {A}^{H})$ are respectively given by

50 $$\begin{aligned} \alpha _{\mathbf {A}}&= \frac{n^{2}}{n^{2}-1} \text {tr}(\mathbf {S}\mathbf {A}\mathbf {S}\mathbf {A}^{H}) - \frac{n}{n^{2}-1} \text {tr}(\mathbf {S}\mathbf {A}) \text {tr}(\mathbf {S}\mathbf {A}^{H}), \end{aligned}$$

51 $$\begin{aligned} \beta _{\mathbf {A}}&= \frac{n^{2}}{n^{2}-1} \text {tr}(\mathbf {S}\mathbf {A}) \text {tr}(\mathbf {S}\mathbf {A}^{H}) - \frac{n}{n^{2}-1} \text {tr}(\mathbf {S}\mathbf {A}\mathbf {S}\mathbf {A}^{H}). \end{aligned}$$

### Proof

By Theorem 2, for an arbitrary nonrandom matrix $\mathbf {A}$, we have

52 $$\begin{aligned} \mathbb {E}\begin{pmatrix} \text {tr}(\mathbf {S}\mathbf {A}\mathbf {S}\mathbf {A}^{H}) \\ \text {tr}(\mathbf {S}\mathbf {A}) \text {tr}(\mathbf {S}\mathbf {A}^{H}) \end{pmatrix} = \begin{pmatrix} 1 &{} \frac{1}{n} \\ \frac{1}{n} &{} 1 \end{pmatrix} \begin{pmatrix} \text {tr}(\mathbf {\Sigma }\mathbf {A}\mathbf {\Sigma }\mathbf {A}^{H}) \\ \text {tr}(\mathbf {\Sigma }\mathbf {A}) \text {tr}(\mathbf {\Sigma }\mathbf {A}^{H}) \end{pmatrix}. \end{aligned}$$

Because

53 $$\begin{aligned} \begin{pmatrix} 1 &{} \frac{1}{n} \\ \frac{1}{n} &{} 1 \end{pmatrix}^{-1} = \frac{1}{n^{2}-1} \begin{pmatrix} n^{2} &{} -n \\ -n &{} n^{2} \end{pmatrix}, \end{aligned}$$

we can obtain

54 $$\begin{aligned} \mathbb {E}\left( \frac{1}{n^{2}-1} \begin{pmatrix} n^{2} &{} -n \\ -n &{} n^{2} \end{pmatrix} \begin{pmatrix} \text {tr}(\mathbf {S}\mathbf {A}\mathbf {S}\mathbf {A}^{H}) \\ \text {tr}(\mathbf {S}\mathbf {A}) \text {tr}(\mathbf {S}\mathbf {A}^{H}) \end{pmatrix} \right) = \begin{pmatrix} \text {tr}(\mathbf {\Sigma }\mathbf {A}\mathbf {\Sigma }\mathbf {A}^{H}) \\ \text {tr}(\mathbf {\Sigma }\mathbf {A}) \text {tr}(\mathbf {\Sigma }\mathbf {A}^{H}) \end{pmatrix}. \end{aligned}$$

Therefore, we have

55 $$\begin{aligned} \mathbb {E}\left[ \frac{n^{2}}{n^{2}-1} \text {tr}(\mathbf {S}\mathbf {A}\mathbf {S}\mathbf {A}^{H}) - \frac{n}{n^{2}-1} \text {tr}(\mathbf {S}\mathbf {A}) \text {tr}(\mathbf {S}\mathbf {A}^{H})\right]&= \text {tr}(\mathbf {\Sigma }\mathbf {A}\mathbf {\Sigma }\mathbf {A}^{H}), \end{aligned}$$

56 $$\begin{aligned} \mathbb {E}\left[ \frac{n^{2}}{n^{2}-1} \text {tr}(\mathbf {S}\mathbf {A}) \text {tr}(\mathbf {S}\mathbf {A}^{H}) - \frac{n}{n^{2}-1} \text {tr}(\mathbf {S}\mathbf {A}\mathbf {S}\mathbf {A}^{H}) \right]&= \text {tr}(\mathbf {\Sigma }\mathbf {A}) \text {tr}(\mathbf {\Sigma }\mathbf {A}^{H}). \end{aligned}$$

This concludes the proof.

By Corollary 4, when $\mathbf {A}= \mathbf {I}_{p}$, the unknown scalar quantities $\text {tr}(\mathbf {\Sigma }^{2})$ and $\text {tr}^{2} (\mathbf {\Sigma })$ are unbiasedly estimated by

57 $$\begin{aligned} \alpha _{\mathbf {I}_{p}}&= \frac{n^{2}}{n^{2}-1} \text {tr}(\mathbf {S}^{2}) - \frac{n}{n^{2}-1} \text {tr}^{2} (\mathbf {S}), \end{aligned}$$

58 $$\begin{aligned} \beta _{\mathbf {I}_{p}}&= \frac{n^{2}}{n^{2}-1} \text {tr}^{2} (\mathbf {S}) - \frac{n}{n^{2}-1} \text {tr}(\mathbf {S}^{2}). \end{aligned}$$

When $\mathbf {A}= \mathbf {L}_{p}$, the unbiased estimates of $\text {tr}(\mathbf {\Sigma }\mathbf {L}_{p} \mathbf {\Sigma }\mathbf {L}_{p}^{H})$ and $\text {tr}(\mathbf {\Sigma }\mathbf {L}_{p}) \text {tr}(\mathbf {\Sigma }\mathbf {L}_{p}^{H})$ are respectively given by

59 $$\begin{aligned} \alpha _{\mathbf {L}_{p}}&= \frac{n^{2}}{n^{2}-1} \text {tr}(\mathbf {S}\mathbf {L}_{p} \mathbf {S}\mathbf {L}_{p}^{H}) - \frac{n}{n^{2}-1} \text {tr}(\mathbf {S}\mathbf {L}_{p}) \text {tr}(\mathbf {S}\mathbf {L}_{p}^{H}), \end{aligned}$$

60 $$\begin{aligned} \beta _{\mathbf {L}_{p}}&= \frac{n^{2}}{n^{2}-1} \text {tr}(\mathbf {S}\mathbf {L}_{p}) \text {tr}(\mathbf {S}\mathbf {L}_{p}^{H}) - \frac{n}{n^{2}-1} \text {tr}(\mathbf {S}\mathbf {L}_{p} \mathbf {S}\mathbf {L}_{p}^{H}). \end{aligned}$$

For $q = -(p-1), \dots , p-1$ and $\mathbf {A}= \mathbf {J}_{q}$, the unbiased estimates of $\text {tr}(\mathbf {\Sigma }\mathbf {J}_{q} \mathbf {\Sigma }\mathbf {J}_{q}^{H})$ and $\text {tr}(\mathbf {\Sigma }\mathbf {J}_{q}) \text {tr}(\mathbf {\Sigma }\mathbf {J}_{q}^{H})$ are respectively given by

61 $$\begin{aligned} \alpha _{\mathbf {J}_{q}}&= \frac{n^{2}}{n^{2}-1} \text {tr}(\mathbf {S}\mathbf {J}_{q} \mathbf {S}\mathbf {J}_{q}^{H}) - \frac{n}{n^{2}-1} \text {tr}(\mathbf {S}\mathbf {J}_{q}) \text {tr}(\mathbf {S}\mathbf {J}_{q}^{H}), \end{aligned}$$

62 $$\begin{aligned} \beta _{\mathbf {J}_{q}}&= \frac{n^{2}}{n^{2}-1} \text {tr}(\mathbf {S}\mathbf {J}_{q}) \text {tr}(\mathbf {S}\mathbf {J}_{q}^{H}) - \frac{n}{n^{2}-1} \text {tr}(\mathbf {S}\mathbf {J}_{q} \mathbf {S}\mathbf {J}_{q}^{H}). \end{aligned}$$

Therefore, the available shrinkage intensity for the target $\mathbf {T}_{1}$ is

63 $$\begin{aligned} {\hat{w}}_{\mathbf {T}_{1}} = 0 \wedge \frac{\beta _{\mathbf {I}_{p}} - \frac{1}{p} \alpha _{\mathbf {I}_{p}} - \frac{4}{p(p-1)} \alpha _{\mathbf {L}_{p}}}{\beta _{\mathbf {I}_{p}} - \frac{1}{p} \alpha _{\mathbf {I}_{p}} - \frac{4}{p(p-1)} \alpha _{\mathbf {L}_{p}} + {\hat{d}}_{\mathbf {T}_{1}}} \vee 1, \end{aligned}$$

where

64 $$\begin{aligned} {\hat{d}}_{\mathbf {T}_{1}} = n \alpha _{\mathbf {I}_{p}} - \frac{n}{p} \beta _{\mathbf {I}_{p}} - \frac{4n}{p(p-1)} \beta _{\mathbf {L}_{p}}. \end{aligned}$$

The corresponding linear shrinkage estimator of $\mathbf {\Sigma }$ is

65 $$\begin{aligned} {\hat{\mathbf {\Sigma }}}_{\mathbf {T}_{1}} = (1 - {\hat{w}}_{\mathbf {T}_{1}}) \mathbf {S}+ {\hat{w}}_{\mathbf {T}_{1}} \mathbf {T}_{1}. \end{aligned}$$

For the target $\mathbf {T}_{2}$, the available shrinkage intensity is

66 $$\begin{aligned} {\hat{w}}_{\mathbf {T}_{2}} = 0 \wedge \frac{\beta _{\mathbf {I}_{p}} - \sum _{q=-(p-1)}^{p-1} \frac{1}{p-|q|} \alpha _{\mathbf {J}_{q}}}{\beta _{\mathbf {I}_{p}} - \sum _{q=-(p-1)}^{p-1} \frac{1}{p-|q|} \alpha _{\mathbf {J}_{q}} + {\hat{d}}_{\mathbf {T}_{2}}} \vee 1, \end{aligned}$$

where

67 $$\begin{aligned} {\hat{d}}_{\mathbf {T}_{2}} = n \alpha _{\mathbf {I}_{p}} - \sum _{q=-(p-1)}^{p-1} \frac{n}{p-|q|} \beta _{\mathbf {J}_{q}}. \end{aligned}$$

The corresponding linear shrinkage estimator is

68 $$\begin{aligned} {\hat{\mathbf {\Sigma }}}_{\mathbf {T}_{2}} = (1 - {\hat{w}}_{\mathbf {T}_{2}}) \mathbf {S}+ {\hat{w}}_{\mathbf {T}_{2}} \mathbf {T}_{2}. \end{aligned}$$

Finally, we compare the expressions of the proposed shrinkage intensities and their existing competitors in Table 1.

**Table 1:** Comparison of shrinkage intensities for Toeplitz-structured target.

References	Target	Intensity expression	Method
Liu^30^	$\mathbf {T}_{2}$	$\frac{(n-3)\text {tr}(\mathbf {S}^2)+(n-1)\text {tr}^2(\mathbf {S})}{(n-2)(n+1)\text {tr}(\mathbf {S}-\mathbf {T}_2)^2} \vee 1$	Complex Gaussian, biased
Tong^31^	$\mathbf {T}_{1}$, $\mathbf {T}_{2}$	$0 \wedge \frac{\frac{n\text {tr}(\mathbf {S}^2)}{n-1} - 2\text {tr}(\mathbf {S}\mathbf {T}) + \text {tr}(\mathbf {T}^2) - \frac{\sum _{i=1}^{n}\Vert \mathbf {x}_i\Vert ^2}{n(n-1)}}{\frac{(n^2-2n)\text {tr}(\mathbf {S}^2)}{(n-1)^2} - 2\text {tr}(\mathbf {S}\mathbf {T}) + \text {tr}(\mathbf {T}^2) + \frac{\sum _{i=1}^{n}\Vert \mathbf {x}_i\Vert ^2}{n(n-1)}} \vee 1$	Distribution free, cross-validation
This paper	$\mathbf {T}_{1}$	$0 \wedge \frac{\beta _{\mathbf {I}_{p}} - \frac{1}{p} \alpha _{\mathbf {I}_{p}} - \frac{4}{p(p-1)} \alpha _{\mathbf {L}_{p}}}{\beta _{\mathbf {I}_{p}} - \frac{1}{p} \alpha _{\mathbf {I}_{p}} - \frac{4}{p(p-1)} \alpha _{\mathbf {L}_{p}} + {\hat{d}}_{\mathbf {T}_{1}}} \vee 1$	Complex Gaussian, unbiased
This paper	$\mathbf {T}_{2}$	$0 \wedge \frac{\beta _{\mathbf {I}_{p}} - \sum _{q=-(p-1)}^{p-1} \frac{1}{p-|q|} \alpha _{\mathbf {J}_{q}}}{\beta _{\mathbf {I}_{p}} - \sum _{q=-(p-1)}^{p-1} \frac{1}{p-|q|} \alpha _{\mathbf {J}_{q}} + {\hat{d}}_{\mathbf {T}_{2}}} \vee 1$	Complex Gaussian, unbiased

## Methods

This section provides some numerical simulations and apply them to array signal processing to verify the performance of the proposed estimators. For the sake of notation, the proposed shrinkage estimators in (65) and (68) are denoted as LST1 and LST2. The optimal shrinkage estimators for targets $\mathbf {T}_{1}$ and $\mathbf {T}_{2}$ are respectively denoted as LSO1 and LSO2. The existing competitor for $\mathbf {T}_2$ in^30^ is denoted as LSZ. The competitors in^31^ for $\mathbf {T}_1, \mathbf {T}_2$ are respectively denoted as LSCV1 and LSCV2.

### Numerical simulations

This subsection verifies the estimation accuracy of the available shrinkage intensities by plugging in the unbiased estimates given in Corollary 4. Then we compare the MSEs of the shrinkage estimators listed in Table 1. In implementation, we generate the sample from the complex Gaussian distribution $\mathcal{CN}\mathcal{}(\varvec{\mathbf {0}}, \mathbf {\Sigma })$, where $\mathbf {\Sigma }$ is the true covariance matrix. In numerical simulations, we consider two covariance models:

1. Model 1: $\mathbf {\Sigma }= \mathbf {\Sigma }_{\eta } + \upsilon \mathbf {\Sigma }_r$, where $\mathbf {\Sigma }_{\eta } = (\sigma _{i j})_{p \times p}$ and $\sigma _{i j} = \eta ^{|i - j|}$.
2. Model 2: $\mathbf {\Sigma }= \mathbf {\Sigma }_{\rho } + \upsilon \mathbf {\Sigma }_r$, where $\mathbf {\Sigma }_{\rho } = (\sigma _{i j})_{p \times p}$ and $\sigma _{i j} = \frac{1}{2} |i - j + 1|^{2 \rho } - |i - j|^{2 \rho } + \frac{1}{2} |i - j - 1|^{2 \rho }$.

Moreover, the model parameters are $\upsilon = 0.8$, $\eta = 0.8$, $\rho = 0.9$, and $\mathbf {\Sigma }_r$ = $\mathbf {\xi }\mathbf {\xi }^T$ with $\mathbf {\xi }$ being uniformly drawn from the interval [0, 1], to result in a Toeplitz-like covariance matrices. The dimension is $p = 100$, the sample size *n* varies from 10 to 100.

**Figure 1:**
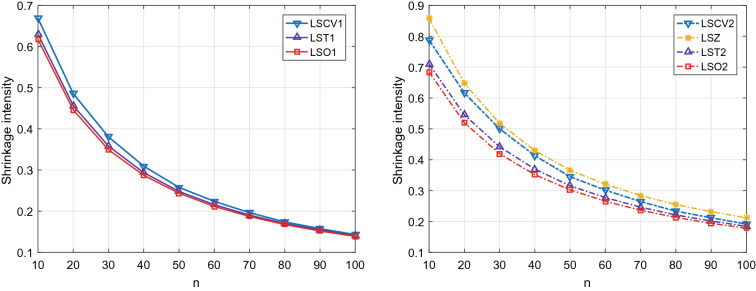
The optimal shrinkage intensity and its estimate for Model 1 with $\eta = 0.8$.

**Figure 2:**
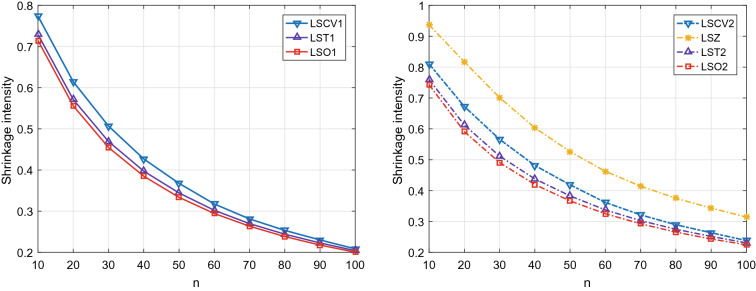
The optimal shrinkage intensity and its estimate for Model 2 with $\rho = 0.9$.

Figures 1 and 2 depict the variation tendencies of the optimal shrinkage intensities and their estimates under Model 1 and Model 2. We can see that the available shrinkage intensities based on the unbiased estimates of unknown scalar quantities are very close to the corresponding theoretical optimal ones. For the target $\mathbf {T}_{1}$, the proposed LST1 outperforms the existing LSCV1 based on the cross-validation method. Moreover, for the target $\mathbf {T}_{2}$, the proposed LST2 performs better than both LSZ based on the biased estimates of unknown scalar quantities and LSCV2 based on the cross-validation method.

Next, we employ the percentage relative improvement in average losses (PRIAL) over the SCM to compare the MSE performance of the proposed covariance estimators and the existing estimators of the same type^14^. The PRIAL is defined by

69 $$\begin{aligned} \mathrm {PRIAL} = \frac{\mathbb {E}[\Vert \mathbf {S}- \mathbf {\Sigma }\Vert ^2] - \mathbb {E}[\Vert {\hat{\mathbf {\Sigma }}} - \mathbf {\Sigma }\Vert ^2]}{\mathbb {E}[\Vert \mathbf {S}- \mathbf {\Sigma }\Vert ^2]}. \end{aligned}$$

The covariance matrix estimator with a larger PRIAL is preferred.

**Figure 3:**
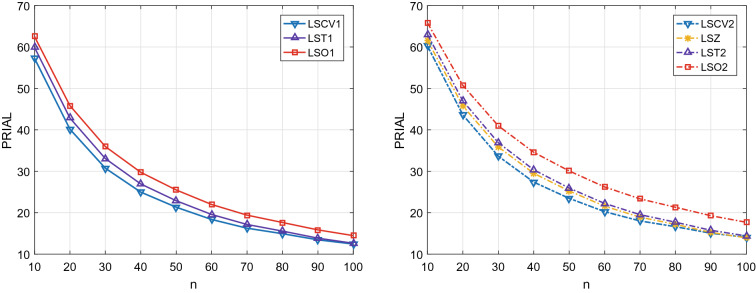
The PRIALs of linear shrinkage estimators for Model 1 with $\eta = 0.8$.

**Figure 4:**
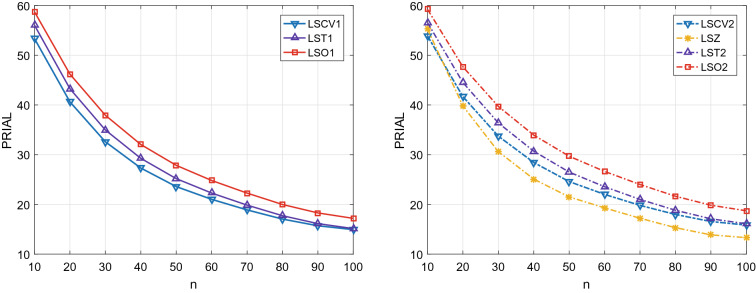
The PRIALs of linear shrinkage estimators for Model 2 with $\rho = 0.9$.

Figures 3 and 4 report the PRIAL values of shrinkage estimators for the two kinds of covariance models, where a larger PRIAL means a smaller MSE. We can see that the PRIALs of proposed LST1 and LST2 are very close to the PRIAL of the optimal shrinkage estimators. When $\mathbf {T}_{1}$ is employed as the target in shrinkage estimation, the proposed LST1 outperforms LSCV1. When $\mathbf {T}_{2}$ is employed, the proposed LST2 outperforms the existing LSZ and LSCV2. Therefore, the proposed covariance matrix estimators for $\mathbf {T}_1$, $\mathbf {T}_2$ enjoy a satisfactory MSE performance.

### Adaptive beamforming

This subsection applies the proposed covariance estimators to array signal processing. In detail, a uniform linear array that consists of sensors with half-wavelength spacing is considered. The received signal at time *t* is

70 $$\begin{aligned} \mathbf {x}(t) = \mathbf {a}(\theta _0) s_0 (t) + \sum _{k=1}^{K} \mathbf {a}(\theta _k) s_k (t) + \mathbf {n}(t) \in \mathbb {C}^p, t = 1, \dots , n, \end{aligned}$$

where $\theta _0$ and $\theta _k$ are respectively the directions of desired complex Gaussian signal $s_0 (t)$ and interference signals $s_k (t)$, $\mathbf {a}(\theta _0)$ and $\mathbf {a}(\theta _k)$ are the corresponding responses, and $\mathbf {n}(t)$ is a white noise^33^. We can obtain the minimum variance distortionless response (MVDR) beamformer:

71 $$\begin{aligned} \mathbf {w}= \frac{\mathbf {\Sigma }^{-1} \mathbf {a}(\theta _0)}{\mathbf {a}(\theta _0)^H \mathbf {\Sigma }^{-1} \mathbf {a}(\theta _0)}. \end{aligned}$$

We usually the covariance matrix $\mathbf {\Sigma }$ in (71) and replace it with its estimate ${\hat{\mathbf {\Sigma }}}$^34^. The corresponding output signal-to-interference-plus-noise ratio (SINR) is

72 $$\begin{aligned} \mathrm {SINR} = \frac{\sigma _0^2 | {\hat{\mathbf {w}}}^H \mathbf {a}(\theta _0) |^2}{{\hat{\mathbf {w}}}^H({\hat{\mathbf {\Sigma }}} - \mathbf {a}(\theta _0) \mathbf {a}(\theta _0)^H){\hat{\mathbf {w}}}}, \end{aligned}$$

where ${\hat{\mathbf {w}}}$ is the realized beamformer based on ${\hat{\mathbf {\Sigma }}}$. The shrinkage estimator with larger output SINR is recommended in array signal processing.

In our experiments, the sensor number is $p = 50$ and the desired signal is assumed to have an angle of arrival of $\theta _0 = 5^{\circ }$ with power $\sigma _0^2 = 10$ dB. There are $K = 3$ interference signals on the directions $-10^{\circ }, 0^{\circ }, 10^{\circ }$ with a same power 8 dB, and an additive complex Gaussian noise with a power 0 dB. For each covariance estimator, we compute the output SINR by averaging $10^{4}$ repetitions.

**Figure 5:**
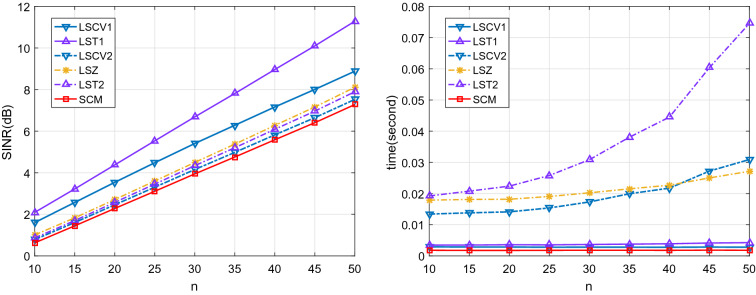
The SINR and corresponding elapsed time of each adaptive beamformer with shrinkage estimator.

Figure 5 reports the SINRs and corresponding elapsed time of adaptive beamformer based on the shrinkage estimators. We can see that the SINR is small when the sample size is small and becomes more significant as the sample size increases. The shrinkage estimators for target $\mathbf {T}_1$ perform better than the ones for target $\mathbf {T}_2$ on SINR. We can see that the target matrix is an important factor in the shrinkage estimation of the covariance matrix. Furthermore, the proposed shrinkage estimator LST1 has the largest SINR and the lowest computational cost, showing that the unbiased estimates of the unknown scalar quantities are conducive to improving the performance of the shrinkage estimator.

### DOA estimation

This subsection investigates the performance of shrinkage estimators in the direction of arrival (DOA) estimation. We assume the linear array consists of $p = 50$ uniformly distributed sensors. There are $K = 5$ mutually independent complex Gaussian source signals from DOA $\mathbf {\theta }= (-10^\circ , -5^\circ , 0^\circ , 5^\circ , 10^\circ )^T$. The signal-to-noise ratio (SNR) is 6 dB. A DOA estimate ${\hat{\mathbf {\theta }}}$ can be obtained using the MUSIC algorithm variant by replacing the SCM with shrinkage estimator^30, 35^. The root mean squared error (RMSE) of DOA estimate ${\hat{\mathbf {\theta }}}$ is defined as

73 $$\begin{aligned} \mathrm {RMSE}({\hat{\mathbf {\theta }}}) = \sqrt{\frac{1}{R K} \sum _{r = 1}^{R} \Vert {\hat{\mathbf {\theta }}}_{r} - \mathbf {\theta }\Vert ^2}, \end{aligned}$$

where *R* is the Monte Carlo run and ${\hat{\mathbf {\theta }}}_{r}$ is the DOA estimate in *r*-th Monte Carlo run.

**Figure 6:**
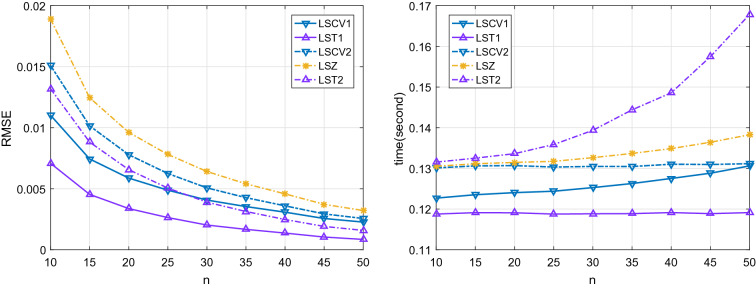
The RMSE and corresponding elapsed time of DOA based on each shrinkage estimator.

Figure 6 reports the RMSEs and corresponding elapsed time of DOA based on the shrinkage estimators. The classic SCM is unavailable in this scenario where $n\le p$. We can see that the estimated error is disappointing as the dimension is large and the sample size is small. The proposed shrinkage estimator LST1 enjoys the lowest RMSE and elapsed time of the others. With an additional time cost, the proposed LST2 can reach a lower RMSE than the existing competitors such as LSZ, LSCV1, and LSCV2 when $n\ge 30$.

## Conclusion

In this paper, we have studied the linear estimation of the covariance matrix in large-dimension and small-sample size scenarios. Under the complex Gaussian distribution, we obtain the optimal shrinkage intensities for the two kinds of Toeplitz-structured targets. By discovering a crucial moment property of the complex Wishart distribution, we unbiasedly estimate the unknown scalar quantities related to the true covariance matrix. Then we propose the shrinkage estimators for the Toeplitz-structured targets. Some numerical simulations and two example applications to signal processing reveal that the proposed covariance matrix estimators dominate the existing estimators in large-dimension and small-sample size scenarios.

In future work, we will continue in-depth studies on the Cramér–Rao bound of the linear shrinkage estimator and investigate nonlinear shrinkage estimation. Moreover, how to choose the target matrix in practice is still a problem worthy of study in shrinkage estimation.

## Data Availability

All data included in this study are available upon request by contact with the corresponding author.
